# Effectiveness of a Web-Based Intervention to Support Medication Adherence Among People Living With HIV: Web-Based Randomized Controlled Trial

**DOI:** 10.2196/17733

**Published:** 2020-04-20

**Authors:** José Côté, Geneviève Rouleau, Maria Pilar Ramirez-Garcia, Patricia Auger, Réjean Thomas, Judith Leblanc

**Affiliations:** 1 Research Centre of the Centre Hospitalier de l’Université de Montréal Montreal, QC Canada; 2 Faculty of Nursing Université de Montréal Montreal, QC Canada; 3 Research Chair in Innovative Nursing Practices Montreal, QC Canada; 4 Clinique médicale l'Actuel Montreal, QC Canada; 5 Sorbonne Université Institut national de la santé et de la recherche médicale Institut Pierre Louis d’Épidémiologie et de Santé Publique Paris France

**Keywords:** medication adherence, people living with HIV, antiretroviral therapy, self-management, nursing, web-based intervention, web-based randomized controlled trial

## Abstract

**Background:**

Taking antiretroviral therapy (ART) is part of the daily life of people living with HIV. Different electronic health (eHealth) initiatives adjunctive to usual care have been proposed to support optimal medication adherence. A web-based intervention called HIV Treatment, Virtual Nursing Assistance, and Education or VIH-TAVIE (from its French version Virus de l’immunodéficience humaine-Traitement assistance virtuelle infirmière et enseignement) was developed to empower people living with HIV to manage their ART and symptoms optimally.

**Objective:**

We aimed to evaluate the effectiveness of VIH-TAVIE in a web-based randomized controlled trial (RCT).

**Methods:**

This RCT was entirely web-based, including recruitment, consent granting, questionnaire completion, and intervention exposure (consultation with VIH-TAVIE [experimental group] or websites [control group]). To be eligible for the study, people living with HIV had to be 18 years or older, be on ART for at least 6 months, have internet access, and have internet literacy. Participants were randomly assigned to either the experimental group (n=45) or control group (n=43). The primary outcome was ART adherence. The secondary outcomes included self-efficacy regarding medication intake, symptom-related discomfort, skills and strategies, and social support. All outcomes were measured with a self-administered web-based questionnaire at the following three time points: baseline and 3 and 6 months later. A generalized linear mixed model was built to assess the evolution of ART adherence over time in both groups.

**Results:**

The sample included 88 participants, and of these, 73 (83%) were men. The median age of the participants was 42 years. Participants had been diagnosed with HIV a median of 7 years earlier (IQR 3-17) and had been on ART for a median of 5 years (IQR 2-12). The proportion of treatment-adherent participants at baseline was high in both groups (34/41, 83% in the experimental group and 30/39, 77% in the control group). Participants also reported high treatment adherence, high self-efficacy, and high skills; perceived good social support; and experienced low discomfort from symptoms. Analyses revealed no intergroup difference regarding ART adherence (OR 1.9, 95% CI 0.6-6.4).

**Conclusions:**

This study highlights the challenges and lessons learned from conducting an entirely web-based RCT among people living with HIV. The challenges were related to the engagement of people living with HIV on the following three levels: starting the web-based study (recruitment), completing the web-based intervention (engagement), and continuing the study (retention). The results contribute to the existing body of knowledge regarding how to conduct web-based evaluation studies of eHealth interventions aimed at developing and strengthening personal skills and abilities.

**Trial Registration:**

ClinicalTrials.gov NCT01510340; https://clinicaltrials.gov/ct2/show/NCT01510340

## Introduction

### Background

Living with HIV means living with a chronic disease that requires taking antiretroviral therapy (ART) for life. It is important to properly coach people living with HIV on this matter in order to encourage them to engage in this health behavior at an optimal level. Various electronic health (eHealth) initiatives adjunctive to the face-to-face services provided by health care teams have been implemented to support people living with HIV in this regard. Daher et al classified eHealth innovations into the following three categories: mobile health-based innovations (essentially SMS text messaging), internet-based mobile innovations (eHealth), and combined innovations (including both SMS text messaging and internet-based eHealth innovations) [1]. Until recently, HIV interventions had been delivered predominantly through SMS text messaging. Systematic reviews and meta-analyses have proved the efficacy of SMS text messaging to enhance treatment adherence [1-4]. Thus, since 2016, the World Health Organization has recommended in its therapy guidelines the inclusion of treatment adherence interventions involving SMS text messaging [5].

In their systematic review covering the period from 1996 to 2017, Daher et al underscored the existence of other less prominent types of internet-based eHealth innovations [1]. These included a two-session computer-delivered motivational intervention to facilitate adherence to newly prescribed ART among youth with HIV [6]; a web-based symptom self-management system for people living with HIV [7]; and a computerized counseling intervention for individuals with adherence problems [8]. At present, research supports the feasibility [7] and efficacy of certain internet-based eHealth innovations to optimize antiretroviral intake [8-10].

Within this context of innovation, we developed a web-based intervention called HIV Treatment, Virtual Nursing Assistance, and Education or VIH-TAVIE (from its French version *Virus de l’immunodéficience humaine-Traitement assistance virtuelle infirmière et enseignement*) to empower people living with HIV to manage their ART and symptoms optimally. VIH-TAVIE consists of four interactive computer sessions (each 20-30 min long) hosted by a virtual nurse who leads the user through a learning process geared to acquiring the requisite skills for treatment adherence. The sessions target self-assessment, motivation, problem solving, emotion regulation, and social skills. These enable people living with HIV to integrate the therapeutic regimen in their everyday routine, manage side effects, and handle problem situations that might interfere with drug intake; interact with health professionals; and mobilize their social network. The development of VIH-TAVIE has been described elsewhere [11]. This web-based nursing intervention is grounded in a disciplinary perspective (the McGill nursing model [12]) and, by extension, in the strength-based approach [13]. Under this model, people and their families are perceived as active participants in health care and learn new ways to cope with the challenges related to the chronic illness. The self-efficacy theory of Bandura was also used [14], particularly to develop skills and strategies to self-manage treatment and symptoms and reinforce one’s self-confidence to take ART.

This web-based tailored nursing intervention demands a certain degree of active engagement on the part of the user in order to develop and strengthen the self-regulatory skills required to deal with difficult situations as they arise. Initially, VIH-TAVIE was evaluated in a hospital setting as an adjunct to conventional care. Participants completed the intervention sessions onsite in a clinical setting. The results of this quasi-experimental study comparing the efficacy of two types of follow-up (conventional vs conventional plus adjunctive web-based sessions [VIH-TAVIE]) in promoting ART adherence among people living with HIV revealed that both groups showed adherence improvement over time but did not differ in this regard [15]. The absence of randomization and a deep selection bias led to the formation of highly heterogeneous groups that limited the scope of the results. Considering the key advantage that web-based tailored interventions afford, namely 24/7 access, we were interested in testing the use of VIH-TAVIE over the internet outside an institutional care setting with a view to reach a broader client group.

Against this background, we conducted a randomized controlled trial (RCT) solely over the internet to test the effectiveness of this web-based intervention for improving and optimizing treatment adherence.

### Study Aim and Hypothesis

The aim of the study was to evaluate the effectiveness of a web-based intervention for optimizing ART adherence among people living with HIV.

Our primary hypothesis was that a higher proportion of participants in the experimental group would show treatment adherence at 6 months (T6) as compared with the control group. Our explanatory hypothesis was that the following variables measured at three time points (baseline [T0], 3 months [T3], and 6 months [T6]) would prove to be mediators capable of explaining the intervention’s effect on treatment adherence: sense of self-efficacy, degree of symptom-related discomfort, skills and strategies used, and perceived social support. These variables are the targets of our intervention [11].

## Methods

### Study Design

A prospective RCT was conducted from February 2012 to September 2017. The study was entirely web-based, including recruitment, consent granting, questionnaire completion, and intervention exposure (consultation with VIH-TAVIE [experimental group] or ART-related websites [control group]).

This RCT is reported according to the CONSORT eHealth Statement [16]. We provide only a brief overview of the study methods, as it has been published elsewhere [17]. The trial has been registered at ClinicalTrials.gov (CE 11.184 / NCT01510340).

### Participants

To be eligible for the study, people living with HIV had to be 18 years or older, be on ART for at least 6 months, have internet access, and be internet literate to be able to complete all web-based procedures by themselves. Participants were recruited via the internet but could have been advised of the study by their health care team and handed a pamphlet with a link to the study’s website. The study was advertised on social networks (Facebook) and on the websites of resources available to people living with HIV, where a hyperlink redirected individuals interested in participating in the web-based research to the study’s website. Recruitment was conducted mainly in the Province of Quebec (Canada). To ensure participants were authentic, we set up validation measures (CAPTCHA authentication and cross validation of sociodemographic variables in the questionnaire).

### Interventions

Participants in the experimental group were invited to consult with VIH-TAVIE that offers four sessions. A 1-week interval was imposed between sessions to ensure the progressive acquisition and consolidation of skills. To encourage participants to complete the next session of the intervention, one email reminder was sent out automatically. Access was thus controlled and predetermined initially. After this period, access to the intervention was unlimited in terms of intensity, frequency, and time of use for the duration of the study. There was no human involvement over the course of the intervention.

Participants in the control group were invited to consult (at their convenience and from the location of their choice) a list of websites offering information on antiretrovirals, their side effects, and their interactions.

### Outcome Measures

The primary outcome was the proportion of ART-adherent participants at T6. Adherence was evaluated by means of a self-administered questionnaire. At the time when the study was planned, there was no clear minimum cutoff point defining what constituted sufficient ART adherence to achieve optimal treatment effectiveness. The cutoff was generally set at greater than 90% or greater than 95% [18]. For this study, optimal adherence was defined as intake of at least 95% of the prescribed tablets in the past 7 consecutive days at T6. The questionnaire was developed and validated among HIV patients [19]. The questionnaire comprised seven items to measure how often a person forgot to take their medication. It was designed to place the respondent in a context where events and situations could lead to lapses.

The secondary outcomes evaluated at T6 are presented below.

Sense of self-efficacy regarding medication intake was measured using 14 items rated on a five-point Likert scale. Two items were added to the original 12-item version used in a previous study [15]. A Cronbach alpha of .92 was obtained for this assessment.

Symptom-related discomfort was measured with an adapted version of the 20-item Self-Completed HIV Symptom Index [20]. Five other items regarding state of health were added to the original 20 items. The 25 items served to determine the presence of symptoms (scale of 0-4, with 0 indicating absent) and degree of discomfort experienced (scale of 1-4). A Cronbach alpha of .89 was obtained for this assessment.

Skills and strategies were measured with a 25-item instrument developed by the research team according to many sub-behaviors required to manage daily antiretroviral treatment over the long term [11]. On a scale of 1 to 5 (1 indicating never and 5 indicating all the time), participants had to gauge how much they used the given skills and strategies. A Cronbach alpha of .92 was obtained for this assessment.

Social support was evaluated using the Medical Outcome Survey [21] and its French version [22]. One dimension of social support was measured with the emotional/informational support subscale, which comprised eight items rated on a five-point Likert scale. The instrument has shown good content validity and appreciable internal consistency [22]. A Cronbach alpha of .96 was obtained for this assessment.

Participants completed a sociodemographic questionnaire covering various characteristics, including gender, age, family situation, education level, annual income, and employment situation, and questions regarding self-perceived state of health, HIV (diagnosis and therapeutic regimen), and immunologic and viral indicators (CD4 cell count and viral load).

All outcomes were measured with a self-administered web-based questionnaire at the following three time points: T0, T3, and T6. Email reminders (maximum of three) were automatically sent out at 7-day intervals prior to measurement.

### Sample Size

The sample size was estimated according to studies by Tuldrà et al [23] and Pradier et al [24] involving people living with HIV and a systematic review by Haynes et al [25] involving adherence-related interventions intended for various groups. To detect a difference of 20 percentage points at 80% power and a chi-square test two-tailed α value of .05, with the benchmark proportion of ART-adherent participants set at 50% and an attrition rate of 20%, the required sample size was 232 participants.

### Randomization and Allocation Concealment

Centralized block balanced randomization in a 1:1 ratio was computer generated. The allocation process was entirely computerized. The participants were informed automatically by email of their group assignment. Only after completion of the baseline questionnaire, participants were randomly assigned to the experimental group (web-based intervention) or control group (general information websites).

### Blinding

Participants were not totally blinded to group assignment. They were aware of randomization to consult a detailed list of websites or complete a web-based nursing intervention. However, the experimental and control groups were not necessarily evident to the participants. During data analysis, the research team was blinded to participant group assignment.

### Statistical Methods

Statistical analyses were based on a per-protocol population and on an intention-to-treat (ITT) population for sensitivity analyses, as recommended in the CONSORT eHealth guidelines [16]. Baseline participant characteristics were reported using frequencies and percentages for categorical variables and medians and IQRs for continuous variables.

The primary outcome was analyzed using the Pearson chi-square test. The Student *t* test (continuous variables) and Pearson chi-square test or Fisher exact test (categorical variables) were used to test for differences in secondary outcomes between the two groups at T6.

In the ITT analysis, participants with missing data at T6 were considered nonadherent.

For exploratory purposes, a generalized linear mixed model (GLMM) with a binomial distribution [26] was built to assess the evolution of the primary outcome over time in both groups in the per-protocol population at T0 (n=80), taking into account hierarchical data and using SAS PROC GLIMMIX (SAS Institute Inc, Cary, North Carolina, USA) [27]. Explanatory variables included strategies used, measurement time points (T0, T3, and T6), and interaction between strategies and time points.

All tests were two-sided, and statistical significance was set at *P*<.05. Analyses were performed using SAS software, version 9.4 (SAS Institute Inc).

### Ethics and Informed Consent

This study was approved by the Research Ethics Board of the Université de Montréal (881) and the Research Centre of the Centre Hospitalier de l’Université de Montréal (11.184). The particularities of the web-based consent procedure have been discussed in detail in the protocol article [17]. Participants were compensated for their participation in the study with a gift certificate of Can $20 after T3 and T6.

## Results

### Participant Characteristics

**Figure 1:**
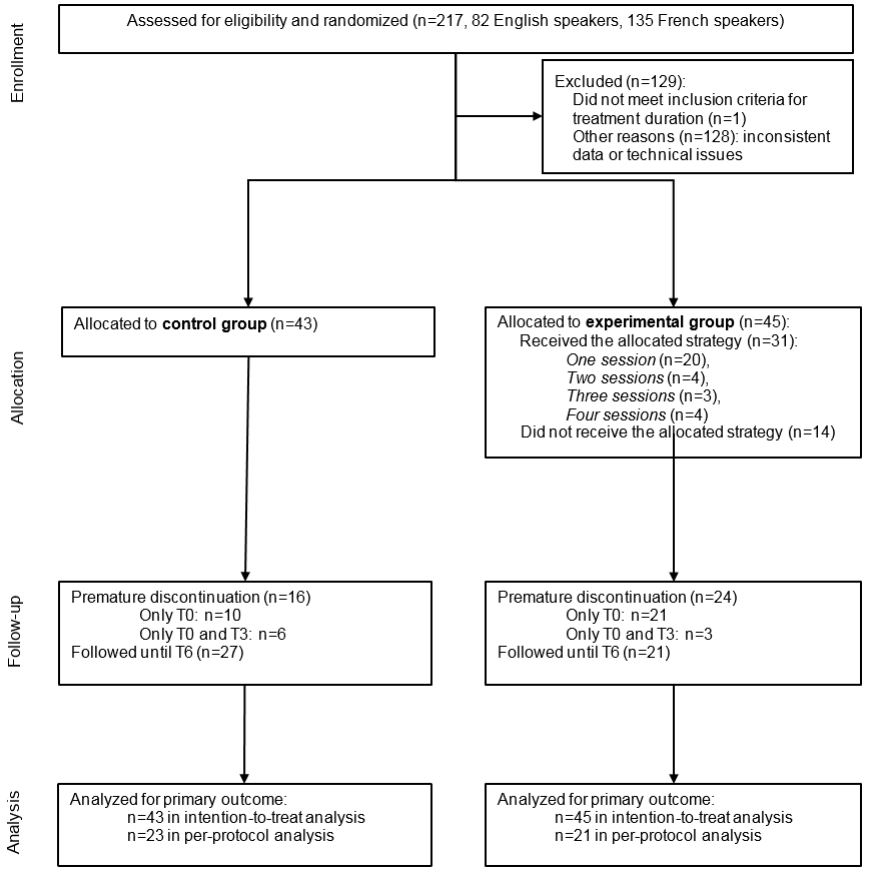
Flow diagram. The measurement time points are baseline (T0) and 3 months (T3) and 6 months later (T6).

Overall, 217 participants were enrolled (Figure 1). Regarding recruitment, the participants were informed about the study primarily by leaflets (32/82, 39%), health care providers (18/82, 22%), and websites (13/82, 15%). One participant was excluded for not meeting the inclusion criteria and 128 were excluded for having inconsistent data. A total of 88 participants were assigned to the experimental group (n=45) or control group (n=43).

### Baseline Sociodemographic and Clinical Data

Baseline sociodemographic and clinical characteristics are described in Table 1. The sample included 13 women and 73 men (two participants with missing data), and the median age of the participants was 42 years (IQR 33-52). In both groups, participants had been diagnosed with HIV a median of 7 years earlier (IQR 3-17) and had been on ART for a median of 5 years (IQR 2-12), with 94% (75/80) declaring an undetectable viral load. Overall, 77% (63/82) of the participants declared homosexual orientation. Further, 56% (44/78) were employed and 72% (57/79) had an annual income greater than Can $15,000. They lived mainly in urban areas. Finally, 94% (82/87) of the participants considered the internet easy or very easy to use and 89% (77/87) used it every day (data not shown).

**Table 1:** Baseline sociodemographic and clinical characteristics of the participants.

Characteristic		Experimental group (N=45), n (%) or median (IQR)		Control group (N=43), n (%) or median (IQR)	
Age, years		43 (33-53)		40 (32-50)	
Male gender^a^		41 (91)		32 (78)	
**Canadian born^b^**		38 (86)		32 (84)	
	Yes		38 (86)		32 (84)
	No		6 (14)		6 (16)
**Marital status^c^**					
	Single		28 (65)		26 (68)
	In a relationship		15 (35)		12 (32)
**Sexual orientation^b^**					
	Heterosexual		8 (18)		9 (24)
	Homosexual		35 (80)		28 (74)
	Bisexual		1 (2)		1 (3)
With children^b^		6 (14)		5 (13)	
HIV-infected children		0 (0)		0 (0)	
**Education level^b^**					
	Primary		0 (0)		0 (0)
	Secondary		9 (21)		13 (34)
	College		11 (25)		14 (37)
	University		24 (55)		11 (29)
**Annual income (in Can $)^d^**					
	<14,999		11 (26)		11 (30)
	15,000-34,999		10 (24)		12 (33)
	35,000-54,999		11 (26)		8 (22)
	>55,000		10 (24)		6 (16)
**Employment status^e^**					
	Employed		27 (64)		17 (47)
	Student		1 (2)		4 (11)
	On welfare		8 (19)		5 (14)
	Others		6 (14)		10 (28)
**Housing/accommodation^c^**					
	Living alone		22 (51)		21 (55)
	Living with spouse		12 (28)		9 (24)
	Living with family or friend		3 (7)		4 (11)
	Others		6 (14)		4 (11)
Self-perceived health (0-10)^e^		8 (7-8)		8 (7-9)	
Years of HIV infection^f^		7 (3-18)		8 (3-16)	
Years of antiretroviral therapy^f^		5 (1-16)		6 (2-10)	
Undetectable viral load^g^		41 (95)		34 (92)	
**CD4 cell count^e^**					
	Did not know		8 (21)		7 (18)
	Knew		30 (79)		33 (83)
Value of CD4 cell count (cells/μl)^h^		555 (410-690)		650 (480-800)	
**CD4 trend^g^**					
	Increasing		8 (19)		11 (30)
	Decreasing		7 (16)		3 (8)
	Stable		16 (37)		18 (49)
	Did not know		12 (28)		5 (14)
Months since last blood control^i^		1 (1-3)		2 (0-3)	
Treatment change in the past 3 months^c^		2 (5)		4 (11)	
**Reasons for change^j^**					
	To switch to more effective drugs		1 (50)		3 (75)
	To reduce adverse events		2 (100)		2 (50)
	To simplify treatment		0 (0)		3 (75)
	Others		1 (50)		0 (0)

^a^Total 86 participants (two missing).

^b^Total 82 participants (six missing).

^c^Total 81 participants (seven missing).

^d^Total 79 participants (nine missing).

^e^Total 78 participants (10 missing).

^f^Total 87 participants (one missing).

^g^Total 80 participants (eight missing).

^h^Total 61 participants (two missing).

^i^Total 85 participants (three missing).

^j^More than one reason possible.

### Attrition and Engagement in the Study Process and Intervention

Of the 88 participants, 48 (55%) completed the questionnaire at T6, with a median of 7 months (IQR 6-8) after baseline, and the attrition rate was 45% (40/88). In terms of engagement in the intervention, in the experimental group, 69% (31/45) of the participants accessed the intervention (Figure 1). Of these participants, 65% (20/31) completed only session one and 36% (11/31) completed more than one session. Among those who complete more than one session, 13% (4/31) completed sessions one and two, 10% (3/31) reached session three, and 13% (4/31) reached session four.

### Primary Outcome

The proportion of treatment-adherent participants (defined as intake of at least 95% of the prescribed tablets in the past 7 consecutive days) at baseline was high, reaching a mean of 80% in both groups (34/41, 83% in the experimental group and 30/39, 77% in the control group). The proportion of treatment-adherent participants at T6 did not differ between the experimental and control groups in the per-protocol analysis (19/21, 91% vs 19/23, 83%; *P*=.67). Results were similar in the ITT analysis (Table 2).

**Figure 2:**
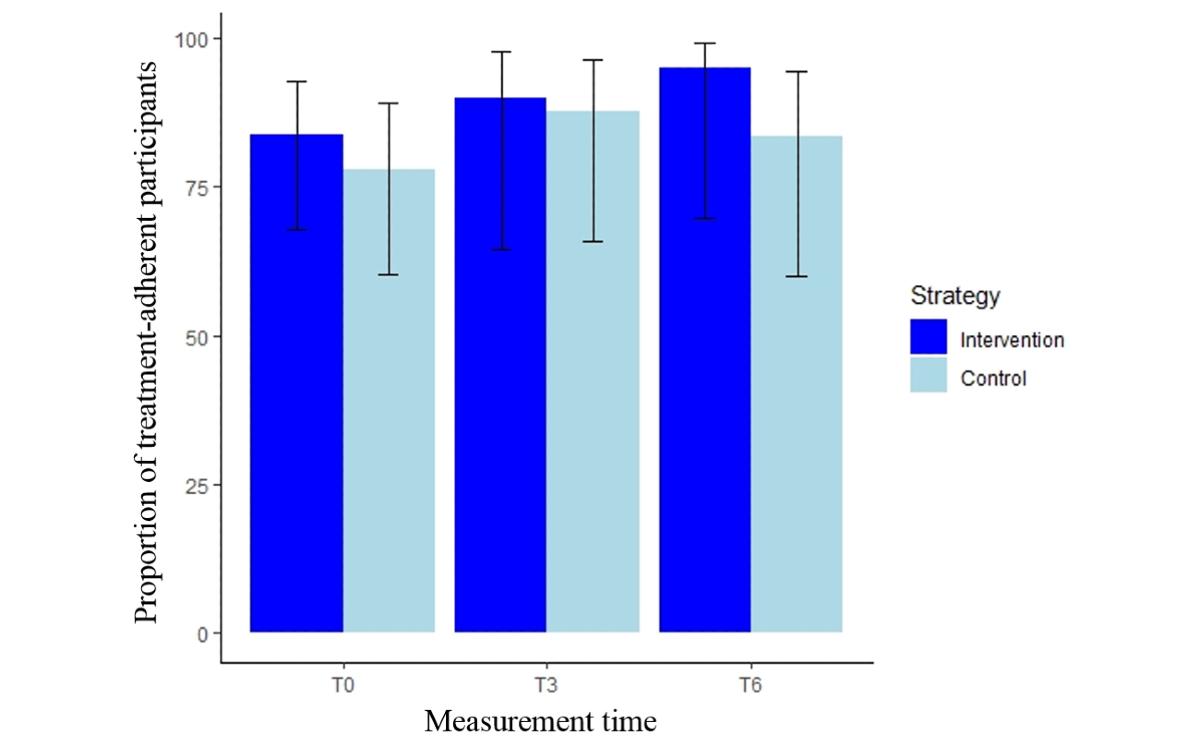
Adherence over time. The solid bars represent the estimated proportion of treatment-adherent participants, and the error bars (lines) indicate the corresponding 95% CIs from the generalized linear mixed model. The measurement time points are baseline (T0) and 3 months (T3) and 6 months later (T6).

Similar results were confirmed in the exploratory analysis using a GLMM. No intergroup difference was observed (OR 1.9, 95% CI 0.6-6.4). No significant time effect (OR 0.4, 95% CI 0.1-1.6 for the proportion of treatment-adherent participants at T0 vs T6; OR 0.8, 95% CI 0.2-3.8 for the proportion at T3 vs T6) and no strategy-by-time interaction effect on treatment adherence were found (Figure 2).

**Table 2:** Proportion of antiretroviral-adherent participants.

Time point		Experimental group					Control group				*P* value
			Total, n		Value, n (%)		Total, n		Value, n (%)		
**Baseline (T0)**											
	Per-protocol analysis		41		34 (83)		39		30 (77)		
	Intention-to-treat analysis		45		34 (76)		43		30 (70)		
**3 months (T3)**											
	Per-protocol analysis		19		17 (90)		25		22 (88)		
	Intention-to-treat analysis		45		17 (38)		43		22 (51)		
**6 months (T6)**											
	Per-protocol analysis^a^		21		19 (91)		23		19 (83)		.67
	Intention-to-treat analysis^a^		45		19 (42)		43		19 (44)		.85

^a^For the primary outcome, groups were compared using the Pearson chi-square test.

### Secondary Outcomes

Table 3 presents a description of the secondary outcomes. At T6, participants reported low discomfort in terms of symptom count or bother, and there was no intergroup difference in this regard. Participants also expressed a high sense of self-efficacy and an elevated level of social support, both of which tended to improve over time. There was again no intergroup difference in this regard. Reported skills and strategies were high at baseline and T6.

**Table 3:** Secondary outcomes at 6 months (T6).

Variable	Experimental group (N=45), median (IQR) score	Control group (N=43), median (IQR) score	*P* value^a^
Symptom count^b^	9.0 (6.0-14.0)	7.5 (5.0-17.5)	.68
Symptom bother^c^	17.0 (11.0-29.0)	18.5 (8.5-47.0)	.70
Self-efficacy^d^	67.5 (60.5-70.0)	66.0 (62.0-69.0)	.81
Social support^e^	32.0 (28.0-39.0)	32.0 (27.0-38.0)	.83
Skills and strategies^f^	97.0 (82.0-118.0)	98.0 (90.0-111.0)	.90

^a^Groups were compared using the Student *t* test or Fisher exact test.

^b^Total 45 participants (three missing). Possible score range 0-25.

^c^Total 45 participants (three missing). Possible score range 1-100.

^d^Total 39 participants (nine missing). Possible score range 14-90.

^e^Total 47 participants (one missing). Possible score range 8-40.

^f^Total 46 participants (two missing). Possible score range 25-125.

## Discussion

The objective of this study was to evaluate the effectiveness of a web-based intervention for optimizing adherence to antiretroviral intake in people living with HIV. The results showed no intergroup difference for treatment adherence. Participants in both the experimental and control groups had been living with HIV for 7 years and had been on ART for 5 years. They self-reported high treatment adherence, high self-efficacy, and high skills; perceived good social support; and experienced low discomfort from symptoms.

These results are comparable to those obtained in our previous study involving people living with HIV frequenting a clinic [15]. However, the participants in this study and our previous study differ in terms of sociodemographic characteristics. The participants in this study were younger (this study vs previous study: 41 years vs 48 years), had been living with HIV for a shorter period of time (7 years vs 11 years), had a higher education level (college or university diploma: 60/82, 73% vs 86/179, 48%), and had a higher income (>Cad $15,000: 57/79, 72% vs 70/179, 39%). Regarding internet literacy, the majority went online every day and considered web navigation easy.

Contrary to our results, Kurth et al found an improvement in self-reported treatment adherence (on a 30-day visual analog scale) among people living with HIV (n=240) exposed to a computerized counseling tool [8]. More specifically, among participants with a nonsuppressed viral load at baseline, adherence increased by about 10% in the experimental group (76% at baseline to 85% 9 months later), whereas in the control group, the rate started at 74% and showed no improvement over time. In other words, the adherence effect was more pronounced among people living with HIV having a detectable viral load. A suppressed viral load was noted in 66% of participants in their sample as compared with more than 90% of participants in our sample (self-reported viral load). We believe that the high rate of adherence and suppressed viral load among participants in our study might have left little room for improvement, unlike that in the study by Kurth et al [8]. Their intervention, which shares similar components with our intervention, is based on Bandura theory and consists of four sessions that include audio-narrated assessment, tailored feedback, skill-building videos, a health plan, and printouts. This intervention, much like VIH-TAVIE, is geared for skill building and patient empowerment. According to a systematic review by Zhang et al, the use of information and communication technology in HIV self-management interventions is an emerging field [28]. They identified the following three major functionalities of such interventions: deliver information modules, support self-monitoring medical adherence, and provide access to HIV self-management information.

To determine treatment adherence, we set the cutoff point at 95%, which was commonly used at the time we planned and conducted our study. However, according to a recent meta-analysis by Bezabhe et al, adherence levels as low as 80% to 90% are good enough to achieve viral suppression [29]. As stated by these researchers, the clinical importance of this finding lies in the fact that the “level of adherence behavior capable of sustaining viral suppression is broader than previously thought.” Considering this, in our sample, it is possible and even plausible that all of the recruited people living with HIV were treatment adherent before being exposed to the intervention. Indeed, they might already have been nearly fully engaged in the adherence behavior and strongly mobilized regarding ART intake, as evidenced by their self-reported high levels of self-efficacy and skills.

Compared with our previous study conducted in a clinical setting with nurses present onsite to facilitate the overall flow of research and the consultation with VIH-TAVIE [15], the present study was entirely web-based, including participant recruitment, consent granting, data collection, and participant follow-up across 6 months. Various challenges emerged relative to this approach of conducting a study that aimed to not only evaluate a web-based intervention but also conduct the evaluation entirely over the internet. According to a literature review by Pham et al, the vast majority of mHealth clinical trials conducted in the past favored onsite study implementation (69/71, 97%). In fact, they found only two web-based trials that recruited and collected data via the internet (2/71, 3%) [30]. Recently, a systematic review (n=41) by Price et al on the quality of web-based self-management trials underscored the challenges related to this type of study and concluded that web-based trials were still an emergent field [31].

In our study, challenges were related to engagement on the following three levels: starting the web-based study, completing the web-based intervention, and continuing the study.

Participant recruitment and engagement to start the study are key stages in the research process. Different modalities must be implemented to reach the target client group and to ensure their participation. In our study, we employed a multimodal strategy of offline and web-based recruitment that involved a mix of traditional and innovative channels, including newspapers, magazines, hospitals, health care providers, free internet methods, and Facebook. However, the majority of participants reported being reached by more traditional methods (61% by leaflets and health care providers). As many authors have pointed out in the past, the importance of cultivating close ties with health care settings is all the more obvious when seeking to reach a client group with a health problem [32]. A strong alliance with the care setting is imperative to ensure the credibility of the proposed approach and intervention, which should be in line with the care delivered in the clinical setting. Indeed, participant engagement in a web-based RCT requires a great deal of motivation that goes beyond an initial interest or curiosity. Millard et al performed a study of the efficacy of a web-based self-management program in improving health outcomes for people living with HIV and revealed that only 58% (132/227) of the participants recruited for the study completed the web-based registration form and baseline questionnaires [33].

Engagement in the intervention is another challenge. Among 69% (31/45) of participants who accessed the intervention, the majority completed only the first session (20/31, 65%). Yet, in our previous study conducted in a clinical care setting, engagement seemed optimal, although participants had to travel to the site. In that case, 74% (73/99) of the participants completed all four VIH-TAVIE sessions and only four participants completed none of the sessions (4/99, 4%) [15]. However, a review by Price et al on the quality of web-based self-management trials evidenced that engagement in interventions over time was not optimal [31]. According to Sieverink et al [34], participants did not use technologies in the desired way most of the time. These researchers raised the following question: Do all users need to experience all of the elements of a technology to obtain effects? In the opinion of Sieverink et al [34], depending on the user’s goals and the desired outcomes, technology could be employed in many different ways in terms of features used, frequency of use, time of use, and place of use. Moreover, individuals might also stop using technology once they reach their personal goals. This sort of dropout was not necessarily a consequence of losing interest. Another important aspect is whether engagement should be measured according to the number of logins, number of sessions completed, or number of pages viewed. According to Sieverink et al [34], the unspoken rule is “the more, the better.” They concluded that adherence to eHealth technology was an underdeveloped and often improperly used concept in the existing body of literature. In the case of our study, given that participants manifested high levels of sense of self-efficacy, skills, and treatment adherence at baseline, it is not unreasonable to think that after the first session, skills were already consolidated and participants had no reason to continue with the intervention.

Participant engagement to see the study through (ie, retention over 6 months) was low (48/88, 55%), indicating that attrition was high at 45% (40/88). In the studies reviewed by Price et al, 73% (30/41) of the web-based trials reported high attrition rates with incomplete or unreported data [31]. To ensure a high rate of retention, Watson et al used intensive follow-up modalities in their web-based RCT in the general population [35]. These modalities were deployed sequentially over time until the survey was completed (web, telephone, mailed survey, and a postcard with selected outcomes). According to these authors, offering bonus incentives and diverse follow-up modalities were key factors contributing to a high rate of data retention. In our study, incentives and email reminders were used to engage and follow the participants. However, intensive follow-up modalities (telephone and mail survey) might be difficult to implement and inappropriate or irrelevant for people living with HIV, given the persistent stigmatization of the illness. Despite their success, Watson et al [35] recognized that obtaining an adequate sample size, keeping participants engaged in the study, and achieving adequate rates of outcome data retention were extremely challenging tasks.

Presently, there are no best-practice standards for recruiting or retaining participants in web-based trials. However, the lack of face-to-face interaction is a major issue in terms of how interventions are delivered [28] and how studies are conducted [31]. Regarding engagement and recruitment relative to digital health interventions, a more hybrid approach (face-to-face and web-based components) appears to be a serious option to consider [32,36]. Still, notwithstanding all these difficulties and challenges, there are advantages to conducting a web-based RCT. It may allow reaching and including people with limited mobility, people in nonurban areas (where the study is not available), and people with stigmatizing conditions (offers greater sense of confidentiality and anonymity). According to Watson et al, this type of study affords a multitude of advantages, including automated data collection and high control over intervention content and format [35]. The use of a comparative intervention constitutes a further strong point of our parallel RCT design. Regarding the study’s limitations, those related to engagement in the intervention and attrition have been discussed in detail above. Despite using a conservative approach to eliminate false participants and ensure data quality, our study may have suffered from selection bias (participants willing to respond over the internet) and reliance on self-reported outcomes. On account of these limitations, Price et al [31] believed that this type of web-based trial is more pragmatic than explanatory trials.

Al-Durra et al revealed that 27% of the results from digital health registered clinical trials had never been published [37]. This is lower than the nonpublication rate in other fields (impact and risk of publication bias in the field of digital health trials) and is attributed to challenges specific to digital heath randomized clinical trials (high attrition rate and usability issues). Despite these limitations, the findings of our study add to the existing body of knowledge regarding how to conduct web-based studies that evaluate eHealth interventions aimed at developing and strengthening personal skills and abilities.

## Appendix

Multimedia Appendix 1 CONSORT-eHEALTH checklist (V 1.6.1).

## Abbreviations

ART: antiretroviral therapy
eHealth: electronic health
GLMM: generalized linear mixed model
ITT: intention-to-treat
RCT: randomized controlled trial
VIH-TAVIE: Virus de l’immunodéficience humaine-Traitement assistance virtuelle infirmière et enseignement (HIV Treatment, Virtual Nursing Assistance, and Education)

## References

[1] Daher J, Vijh R, Linthwaite B, Dave S, Kim J, Dheda K, Peter T, Pai NP (2017). Do digital innovations for HIV and sexually transmitted infections work? Results from a systematic review (1996-2017). BMJ Open.

[2] Amankwaa I, Boateng D, Quansah DY, Akuoko CP, Evans C (2018). Effectiveness of short message services and voice call interventions for antiretroviral therapy adherence and other outcomes: A systematic review and meta-analysis. PLoS One.

[3] Cooper V, Clatworthy J, Whetham J, Consortium E (2017). mHealth Interventions To Support Self-Management In HIV: A Systematic Review. Open AIDS J.

[4] Quintana Y, Gonzalez Martorell EA, Fahy D, Safran C (2018). A Systematic Review on Promoting Adherence to Antiretroviral Therapy in HIV-infected Patients Using Mobile Phone Technology. Appl Clin Inform.

[5] World Health Organization (2013). Consolidated Guidelines On The Use Of Antiretroviral Drugs For Treating And Preventing Hiv Infection: Recommendations For A Public Health Approach.

[6] Naar-King S, Outlaw AY, Sarr M, Parsons JT, Belzer M, Macdonell K, Tanney M, Ondersma SJ, Adolescent Medicine Network for HIV/AIDS Interventions (2013). Motivational Enhancement System for Adherence (MESA): pilot randomized trial of a brief computer-delivered prevention intervention for youth initiating antiretroviral treatment. J Pediatr Psychol.

[7] Schnall R, Wantland D, Velez O, Cato K, Jia H (2014). Feasibility testing of a web-based symptom self-management system for persons living with HIV. J Assoc Nurses AIDS Care.

[8] Kurth AE, Spielberg F, Cleland CM, Lambdin B, Bangsberg DR, Frick PA, Severynen AO, Clausen M, Norman RG, Lockhart D, Simoni JM, Holmes KK (2014). Computerized counseling reduces HIV-1 viral load and sexual transmission risk: findings from a randomized controlled trial. J Acquir Immune Defic Syndr.

[9] Hersch RK, Cook RF, Billings DW, Kaplan S, Murray D, Safren S, Goforth J, Spencer J (2013). Test of a web-based program to improve adherence to HIV medications. AIDS Behav.

[10] Fisher JD, Amico KR, Fisher WA, Cornman DH, Shuper PA, Trayling C, Redding C, Barta W, Lemieux AF, Altice FL, Dieckhaus K, Friedland G, LifeWindows T (2011). Computer-based intervention in HIV clinical care setting improves antiretroviral adherence: the LifeWindows Project. AIDS Behav.

[11] Côté J, Godin G, Garcia PR, Gagnon M, Rouleau G (2008). Program development for enhancing adherence to antiretroviral therapy among persons living with HIV. AIDS Patient Care STDS.

[12] Gottlieb L, Rowat K (1987). The McGill model of nursing: a practice-derived model. ANS Adv Nurs Sci.

[13] Gottlieb LN (2012). Strengths-based Nursing Care: Health And Healing For Person And Family.

[14] Bandura A (1997). Self-efficacy: The exercise of control.

[15] Côté J, Godin G, Ramirez-Garcia P, Rouleau G, Bourbonnais A, Guéhéneuc Y, Tremblay C, Otis J (2015). Virtual intervention to support self-management of antiretroviral therapy among people living with HIV. J Med Internet Res.

[16] Eysenbach G, CONSORT- E (2011). CONSORT-EHEALTH: improving and standardizing evaluation reports of Web-based and mobile health interventions. J Med Internet Res.

[17] Côté J, Godin G, Guéhéneuc Y, Rouleau G, Ramirez-Garcìa P, Otis J, Tremblay C, Fadel G (2012). Evaluation of a real-time virtual intervention to empower persons living with HIV to use therapy self-management: study protocol for an online randomized controlled trial. Trials.

[18] Bangsberg DR (2006). Less than 95% adherence to nonnucleoside reverse-transcriptase inhibitor therapy can lead to viral suppression. Clin Infect Dis.

[19] Godin G, Gagné C, Naccache H (2003). Validation of a self-reported questionnaire assessing adherence to antiretroviral medication. AIDS Patient Care STDS.

[20] Justice AC, Holmes W, Gifford AL, Rabeneck L, Zackin R, Sinclair G, Weissman S, Neidig J, Marcus C, Chesney M, Cohn SE, Wu AW, Adult AIDS Clinical Trials Unit Outcomes Committee (2001). Development and validation of a self-completed HIV symptom index. J Clin Epidemiol.

[21] Sherbourne CD, Stewart AL (1991). The MOS social support survey. Social Science & Medicine.

[22] Anderson D, Bilodeau B, Deshaies G, Gilbert M, Jobin J (2005). [French-Canadian validation of the MOS Social Support Survey]. Can J Cardiol.

[23] Tuldrà A, Fumaz CR, Ferrer MJ, Bayés R, Arnó A, Balagué M, Bonjoch A, Jou A, Negredo E, Paredes R, Ruiz L, Romeu J, Sirera G, Tural C, Burger D, Clotet B (2000). Prospective randomized two-Arm controlled study to determine the efficacy of a specific intervention to improve long-term adherence to highly active antiretroviral therapy. J Acquir Immune Defic Syndr.

[24] Pradier C, Bentz L, Spire B, Tourette-Turgis C, Morin M, Souville M, Rebillon M, Fuzibet J, Pesce A, Dellamonica P, Moatti J (2003). Efficacy of an educational and counseling intervention on adherence to highly active antiretroviral therapy: French prospective controlled study. HIV Clin Trials.

[25] Haynes RB, Ackloo E, Sahota N, McDonald HP, Yao X (2008). Interventions for enhancing medication adherence. Cochrane Database Syst Rev.

[26] Molenberghs G, Verbeke G, Springer Science & Business Media (2006). Models for Discrete Longitudinal Data. Part IV. Subject-specific Models.

[27] (2008). SAS/STAT 9.2 User’s Guide.

[28] Zhang Y, Li X (2017). Uses of information and communication technologies in HIV self-management: A systematic review of global literature. International Journal of Information Management.

[29] Bezabhe WM, Chalmers L, Bereznicki LR, Peterson GM (2016). Adherence to Antiretroviral Therapy and Virologic Failure: A Meta-Analysis. Medicine (Baltimore).

[30] Pham Q, Wiljer D, Cafazzo JA (2016). Beyond the Randomized Controlled Trial: A Review of Alternatives in mHealth Clinical Trial Methods. JMIR Mhealth Uhealth.

[31] Price A, Vasanthan L, Clarke M, Liew SM, Brice A, Burls A (2019). SMOOTH: Self-Management of Open Online Trials in Health analysis found improvements were needed for reporting methods of internet-based trials. J Clin Epidemiol.

[32] O'Connor S, Hanlon P, O'Donnell CA, Garcia S, Glanville J, Mair FS (2016). Understanding factors affecting patient and public engagement and recruitment to digital health interventions: a systematic review of qualitative studies. BMC Med Inform Decis Mak.

[33] Millard T, Agius PA, McDonald K, Slavin S, Girdler S, Elliott JH (2016). The Positive Outlook Study: A Randomised Controlled Trial Evaluating Online Self-Management for HIV Positive Gay Men. AIDS Behav.

[34] Sieverink F, Kelders SM, van Gemert-Pijnen JEWC (2017). Clarifying the Concept of Adherence to eHealth Technology: Systematic Review on When Usage Becomes Adherence. J Med Internet Res.

[35] Watson NL, Mull KE, Heffner JL, McClure JB, Bricker JB (2018). Participant Recruitment and Retention in Remote eHealth Intervention Trials: Methods and Lessons Learned From a Large Randomized Controlled Trial of Two Web-Based Smoking Interventions. J Med Internet Res.

[36] Santarossa S, Kane D, Senn CY, Woodruff SJ (2018). Exploring the Role of In-Person Components for Online Health Behavior Change Interventions: Can a Digital Person-to-Person Component Suffice?. J Med Internet Res.

[37] Al-Durra M, Nolan RP, Seto E, Cafazzo JA, Eysenbach G (2018). Nonpublication Rates and Characteristics of Registered Randomized Clinical Trials in Digital Health: Cross-Sectional Analysis. J Med Internet Res.

